# Spermidine enhances heat tolerance of rice seeds during mid-filling stage and promote subsequent seed germination

**DOI:** 10.3389/fpls.2023.1230331

**Published:** 2023-09-18

**Authors:** Yutao Huang, Gaofu Mei, Dongdong Cao, Yebo Qin, Liu Yang, Xiaoli Ruan

**Affiliations:** ^1^ Institute of Crop and Nuclear Technology Utilization, Zhejiang Academy of Agricultural Sciences, Hangzhou, China; ^2^ Zhejiang Agricultural Technology Extension Center, Hangzhou, China; ^3^ Zhejiang Nongke Seed Co.Ltd, Hangzhou, China

**Keywords:** heat stress, rice, seed development, seed germination, spermidine, starch, antioxidant defense

## Abstract

**Introduction:**

Heat stress is a vital factor which restricts rice seed quality and yield. However, the response mechanism to heat stress in the mid filling stage of rice seed is unclear.

**Methods:**

In the present study we integrated phenotypic analysis with biochemical, hormone, and gene expression analysis in order to explore technologies for improving rice seeds heat tolerance and subsequent seed germination.

**Results:**

Spermidine (Spd) application effectively alleviated the damage of heat stress treatment during mid-filling stage (HTM, 12-20 days after pollination) on seed development, promoted subsequent seed germination and seedlings establishment. Spd significantly increased seed dry weight, starch and amylose contents during seed development under heat stress, and improved seed germinate, seedlings establishment and seedling characteristics during germination time. Biochemical analysis indicated that, HTM significantly decreased the activities of several starch synthase enzymes and led to a decrease in starch content. While Spd treatment significantly enhanced the activities of ADP-glucose pyrophosphorylas and granule-bound starch synthase, as well as the corresponding-genes expressions in HTM rice seeds, resulting in the increases of amylose and total starch contents. In addition, Spd significantly increased the catalase and glutathione reductase activities together with corresponding-genes expressions, and lowered the overaccumulation of H2O2 and malondialdehyde in HTM seeds. In the subsequent seed germination process, HTM+Spd seeds exhibited dramatically up-regulated levels of soluble sugars, glucose, ATP and energy charges. Consistently, HTM+Spd seeds showed significantly increased of α-amylose and α-glucosidase activities as well as corresponding-genes expressions during early germination. Moreover, HTM evidently increased the abscisic acid (ABA) content, decreased the gibberellin (GA) content, and accordingly significantly declined the GA/ABA ratio during early rice seeds germination. However, Spd treatment did not significantly affect the metabolism of GA and ABA in seed germination stage.

**Discussion:**

The present study suggested that Spd treatment could effectively alleviate the negative impact of HTM on seed development and the subsequent seed germination, which might be closely correlated with starch synthesis and antioxidant defense during seed filling period, starch decomposition and energy supply in seed germination period.

## Introduction

Rice is planted globally as a critical staple food (Valipour et al., 2014). Rice originated from tropical atmosphere and subtropics, and the extreme high temperature will influence its yield and quality. Under global warming, heat stress frequently occurred in China, which had become one of the major catastrophic climatic factors for rice production (Xiong et al., 2016; Zhao et al., 2017; Yang et al., 2020). It was estimated that, the rice yield will decrease by 41% by the end of the 21st century due to the frequent heat stress (Peng et al., 2004). Meanwhile, heat stress will also impair the rice seed quality. Heat stress during the rice seed filling increased the chalkiness of rice seeds, and lower the thousand seed weight and germination rate, thereby severely affecting the production of rice seed (Tang et al., 2017). Heat stress has become the bottleneck that restricts rice production in the southern rice region of China. However, the research on the heat stress effect on rice seed development is still lacking. It is worth noting that the reports concerning the heat stress effect on rice seed development mainly concentrates on the flowering and early seed development stage (Madan et al., 2012; Fu et al., 2019; Chen et al., 2021). Nevertheless, severe heat stress frequently occurs during rice seed mid-filing stage (august to october) in the major rice production areas in yangtze river basin of China (including Hubei, Anhui, Jiangxi, Jiangsu and Hunan provinces) (Zhang et al., 2018). Notably, the seed mid-filling stage is a key period for seed vigor formation (Iwai et al., 2012; Martínez-Eixarch and Ellis, 2015; Zhu et al., 2016). Accordingly, investigating the heat resistance mechanism during seed mid-development stage, mitigating the heat stress damage to improve the seed quality are of great practical significance for rice production in China. Starch accumulates in rice endosperm and offers energy to achieve seed germination and seedling establishment; Besides, rice provide over 22% of global energy intake to humans (Tabassum et al., 2021). Starch is comprised by two D-glucose homopolymers, the branched amylopectin and linear amylose. Starch accumulated in rice consists of about 1/4 of amylose and 3/4 of amylopectin (Zhu et al., 2021). Starch biosynthesis represents the complicated biochemical process involving the reactions of various enzymes. Generally, ADP-glucose pyrophosphorylas (ADPGase), soluble starch synthases (SSs), granule-bound starch synthase (GBSS), starch branching enzyme (SBE), and starch debranching enzyme (DBE) are critical starch biosynthesis-related enzymes in higher plants, which are widely investigated in detail (Cai et al., 2006; Huang et al., 2021). Amylose synthesis from glucosyl monomers is catalyzed by GBSS. Starch has the highest abundance in seed endosperm during cereal seed development. Heat stress significantly suppressed starch synthesis during rice seed development. Yamakawa and Hakata (2010) reported that the activities of several AGPases isoforms were down-regulated in Nipponbare rice seeds under heat stress. Heat stress significantly lowered the *OsGBSSI* expression and amylose content of rice seeds during late filling stage (Lin et al., 2010). During rice seed early-filling process, high temperature resulted in the decreased expressions of *SBEI and SBEII*, whereas the increased expression of *SBEIV* (Su et al., 2009). However, the effects of high temperature on starch metabolism during mid-filling process remains to be further explored. Starch degradation to soluble sugars plays a crucial role in supporting seed germination and early seedlings growth. Two enzymic routes for starch degradation had been elucidated in cereals, hydrolysis with amylase and phospholysis with starch phosphorylase. Wang et al. (2022) reported that hydrolysis, rather than phosphorlysis, is the main process for cereal starch degradation during seed germination. It was found that α-amylase was crucial in the starch hydrolysis during rice and maize seeds germination (Zhao and Wang, 2001). It was well known that α-amylase expression in germinating cereal grains was regulated by phytohormone. As discovered by Kim et al. (2006), gibberellic acid (GA) promoted the *de novo* α-amylase synthesis within aleurone layer cells.

Polyamines (PAs) are small aliphatic polycationic nitrogenous compounds, mainly including putrescine (Put), spermidine (Spd) and spermine (Spm) (Liu et al., 2015). Spd is the major triamine, and proved be crucial in regulating plant growth and development, such as flowers differentiation, fruits development and senescence, and seed germination (Carolina et al., 2015; Kamrun et al., 2016; Tao et al., 2018; Alcázar et al., 2020). Spd also plays a vital role in the plant response to heat stress (Ma et al., 2020; Zhang L et al., 2017; Sagor et al., 2013). Spd application mitigated the plant heat damage by inducing the antioxidases defense, maintaining cell membrane stability and stabilizing the photosynthetic system function (Zhang L et al., 2017). Sang et al. (2017) found that exogenous Spd increased the heat tolerance of tomato seedlings through inducing defensive response, protein folding and protein degradation. The overexpression of Spm synthase gene (*AtSPMS*) induced the expression of heat shock factor (*HSF*) and heat shock protein (*HSP*) to mitigate the heat damage to *Arabidopsis thaliana* seedlings (Sagor et al., 2013). Besides, the overexpression of Spd synthase gene (*AtSPDS*) in *Arabidopsis thaliana* up-regulated the expressions of several key stress-response factors (including *WRKY*, *bZIP* and *rd29A*), thereby enhancing the plant resistance to high temperature, drought and flood stress (Kasukabe, 2004). During seed filing process, the endogenous Spd in rice and maize seeds were extremely significantly positively correlated with the seed weight (Wang et al., 2017). Under heat stress, the endogenous Spd in heat-resistant rice varieties and superior grains were more stable than those in heat-sensitive varieties and inferior grains (Chen et al., 2010). Moreover, exogenous Spd induced the expressions of stress-associated proteins (*SAPs*) in the seed early-development stage, and alleviated the heat damage to the hybrid rice seed quality (Fu et al., 2019). The above reports suggested that Spd played an important role in the plant response to heat stress.

However, the study regarding the molecular mechanism of Spd involvement in seed development and seed vigor formation of rice with heat stress treatment during mid-filling stage (HTM) is still lacking. Therefore, the present study aims to explore the potential role of Spd in the regulation of metabolism involved in seed weight accumulation, seed vigor formation and heat stress response process, and elucidate further the mechanism of Spd promoting the seed development of rice.

## Materials and methods

### Materials

Rice seeds of ‘Zhegeng 100’ (*Oryza sativa* L. *ssp*. *japonica*) were used in present study, which has been widely planted in Zhejiang province due to its high yield, better flavor and wide adaptability. The rice plants were grown under normal conditions at the experimental farm of Zhejiang Academy of Agricultural Science (Hangzhou, China). At 8-12 days after pollination (DAP), 10 mL of 0.5 mM Spd rice plants on the spikelets per plant every day for 5 days continuously. The Spd concentration were determined by preliminary experiments. Plants sprayed with distilled water were used as the control. Thereafter, rice plant was placed in the high-temperature growth chambers under the 16-h/8-h light (40°C)/dark (30°C) photoperiod (60% relative humidity) at 12-20 DAP (HTM). Meanwhile, plants grown under16-h/8-h light (30°C)/dark (20°C) photoperiod (60% relative humidity) served as controls (NT). All rice plants were placed to normal environment under 16-h/8-h light (30°C)/dark (20°C) photoperiod at 20-28 DAP.

### Determination of seed characteristics

Seeds dry weight was recorded after 24-h drying under 80°C at 12, 16, 20 and 28 DAP, respectively. Rice seeds were sampled at 28 DAP for determination of seed length, width and thickness using the vernier caliper.

### Seed germination and seedling establishment tests

Rice seeds sampled at 28 DAP were dried to target moisture (14%) at room temperature and then used for seed germination and seedling establishment tests (n=100 under each treatment in four replicates). Seed germination was performed within the germination chambers under the 8-h/16-h light/dark cycle and 25°C conditions. Seed germination was determined in the case of radicle reaching 2/3 of seed length. The germinated seed number was measured daily. Seeds samples were collected at 1, 3, and 5 days of germination for subsequent analyses.

For seedling establishment test, 100 rice seeds were sowed in sands under 25°C and 12-h/12-h light/dark cycle conditions. After 14 days, seedling emergence rate, seedlings height, seedling dry weight, and total chlorophyll content were analyzed in line with experimental requirements.

### Amylose and amylopectin content analysis by dual-wavelength spectrophotometry

Amylose and amylopectin extraction from seed was determined by dual-wavelength spectrophotometry according to the method of Zhu et al. (2021). Briefly, 0.1 g seed starch sample was dissolved in 0.1 mL absolute alcohol and 1 mL sodium hydroxide at 100°C. The mixture was diluted to 10 mL with distilled water. Afterwards, 0.5 mL mixture was sampled and mixed with 0.42 mL 0.1 M hydrochloride solution, 1.08 mL distilled water and 0.1 mL iodine solution, followed by 10 min incubation. The amylose content was determined by the sample absorbance at 607 nm and 433 nm. The amylopectin content was determined by sample absorbance at 729 nm and 543 nm.

### Assay of starch, antioxidant enzymes, PAs metabolism-related enzymes activity

The enzyme activity analysis of ADP-glucose pyrophosphorylase (AGPase), soluble starch synthases (SSs), granule-bound starch synthase (GBSS), starch branching enzyme (SBE), starch debranching enzyme (DBE), α-amylase, β-amylase, α-glucosidase, superoxide dismutase (SOD), catalase (CAT), peroxidase (POD), ascorbate peroxidase (APX), and glutathione reductase (GR) were performed using enzyme-linked immune kit (Mlbio, Shanghai, China). The color change was determined by spectrophotometry using an enzyme mark instrument at the 450 nm wavelength. The enzyme activity was determined through comparison of sample O.D. to standard curve.

### Soluble sugar and glucose contents analysis

Anthrone-H_2_SO_4_ colorimetry was conducted to determine total soluble sugar level of rice seeds (Zhu et al., 2016). In brief, 0.5 g seed sample was dissolved in 10 mL distilled water. The mixture was transformed to 30 mL test tube with stopper, followed by 30 min extraction with at 100°C, dilution till 25 mL with distilled water. The soluble sugar content was determined through comparison of sample absorbance at 620 nm to standard curve.

The glucose content of rice seeds was determined by high performance liquid chromatography (HPLC) with the method of Bailly et al. (2010). In brief, 10 μl glucose extraction was injected into the Spherisorb-NH_2_ column (Thermo Separation Products, France), followed by elution using 75/25 (v/v) acetonitrile/H_2_O_2_ with the Spectra Physics 8700 pump at the 0.8 ml·min^-1^ flow rate. Glucose contents were analyzed through comparison of sample peak area to standard curve.

### ATP and energe charge analysis

ATP and energe charge were determined by HPLC, as described by Liu et al. (2006) with minor modifications (Liu et al., 2006). ATP in the samples were identified by comparison with retention time of standards, while the concentrations of ATP were determined using the external standard method. Data of ATP and energy charge analysis were expressed as means of four replicate determinations.

### Real-Time Quantitative PCR (RT-qPCR)

Total sample RNA was extracted with RNeasy Mini Kit (HuaYueYang, Beijing, China). Seeds RNA (500 ng) was reverse-transcribed into cDNA with PrimeScript RT reagent Kit (Takara, Dalian, China). Gene-specific primers were list in the **Supplementary Table 1**. Briefly, the 20-μl reaction system was used in PCR amplifications, which included 1 μl diluted cDNA, 0.8 μl primers, 10 μl AceQ qPCR SYBR Green Master Mix (Vazyme, Nanjing, China) and 8.2 μl ddH_2_O. Three biological replicates were conducted and each biological replicate was technically repeated three times. All data were expressed as the mean SD after normalization.

### Statistical analysis

Data were analyzed by Statistical Analysis System (SAS) software through analysis of variance (ANOVA). The multiple comparison for mean values were performed by Tukey’S Honestly Significant Difference (HSD) test (*P*<0.05). Prior to ANOVA, percentage data was converted in line with y = arcsin [sqrt (x/100)].

## Results

### Spermidine treatment increased seeds characteristics of HTM during rice seed development

During rice seed development, the seed fresh and dry weight rapidly increased at 12-20 DAP (**Supplementary Figure 1**). Accordingly, heat stress treatment was performed at 12-20 DAP to explore the heat tolerance mechanism of rice during the middle stage of seed filling.

**Figure 1:**
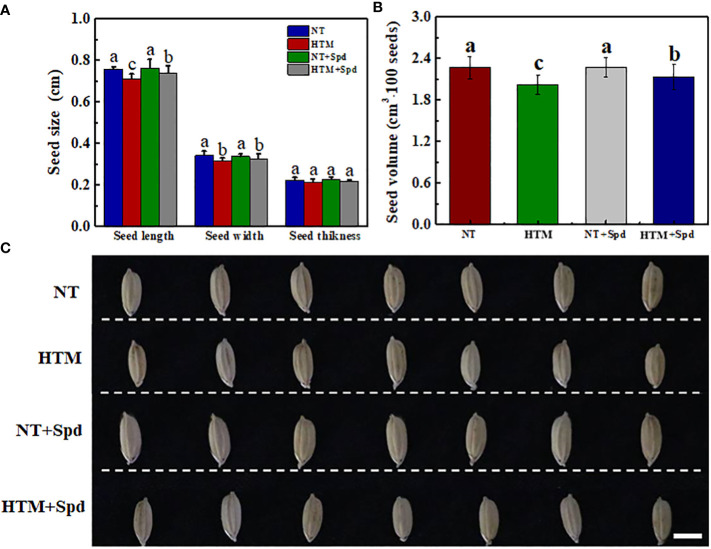
Effects of exogenous Spd on seed characteristics in rice under heat stress. **(A)** Seed size; **(B)** Seed volume; **(C)** Typical images of rice seeds. Seeds were sampled at 28 days after pollination time. NT, normal temperature + distilled water treatment; HTM, heat stress treatment + distilled water treatment; NT + Spd, normal temperature + 0.5 mM Spd treatment; HTM + Spd, heat stress +0.5 mM Spd treatment. Rice plants were treated with Spd solution during 8-12 days after pollination. Heat stress treatment was performed at 12-20 days after pollination. Scale bar, 5 mm. Different lowercase(s) above the bars indicate significant differences (p< 0.05, Tukey’s HSD) among treatments.

At 28 DAP, the seed length, width and volume of HTM seeds were significantly lower than those of NT (**Figure 1**). While HTM+Spd seeds showed significant higher seed length, seed width and volume as compared with HTM seeds. Moreover, Spd treatment markedly increased rice seeds dry weight at 12, 16, and 28 DAP, respectively (**Table 1**). While Spd treatment made no significantly difference on seed characteristics in NT seeds.

**Table 1:** Effects of exogenous Spd on seed dry weight in rice under heat stress.

Treatment	Seeds dry weight (g/1000 seeds)			
	DAP 12	DAP 16	DAP 20	DAP 28
NT	6.87 ± 0.482a*	18.25 ± 1.121a	20.33 ± 1.253a	24.39 ± 1.177a
HTM	6.57 ± 0.451a	15.31 ± 1.113c	17.37 ± 1.034c	21.59 ± 2.022c
NT+Spd	6.66 ± 0.358a	18.87 ± 1.343a	20.57 ± 1.387a	24.18 ± 1.023a
HTM+Spd	6.74 ± 0.471a	17.68 ± 0.929b	18.35 ± 1.993b	23.18 ± 1.542b

*Values followed by a different letter within a column are significantly different at the 0.05 probability level. NT: normal temperature + distill water treatment; HTM: heat stress treatment + distill water treatment; NT+Spd: normal temperature+0.5 mM Spd treatment; HTM+Spd: heat stress +0.5 mM Spd treatment. Rice plants were treated with Spd solution during 8-12 days after pollination. Heat stress treatment was application at 12-20 days after pollination. DAP, days after pollination.

### Spd treatment during rice seed development under heat stress improved subsequent seed germination and seedling emergence

**Figure 2:**
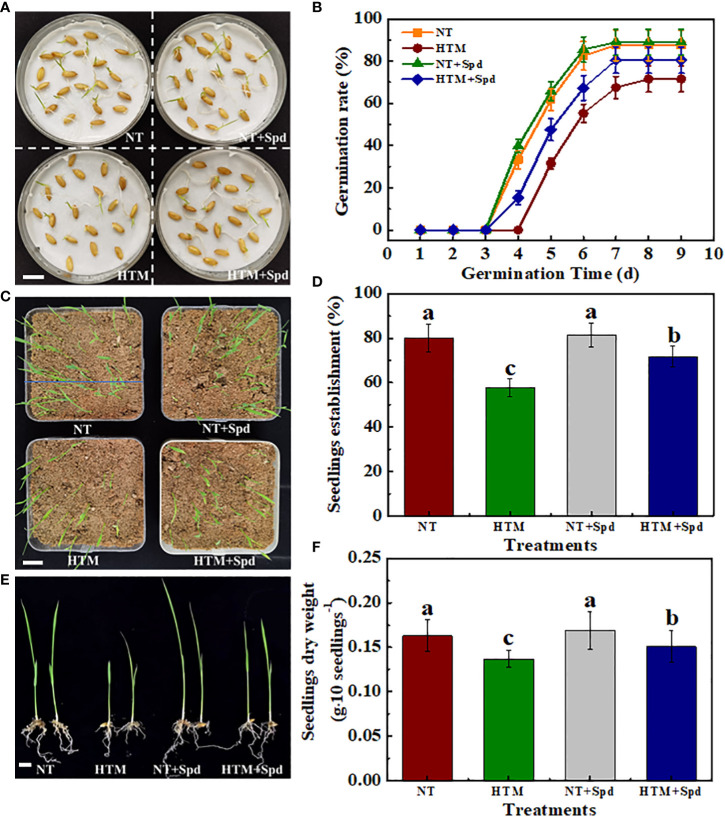
Spermidine treatment remarkably promoted seed germination and seedling emergence of HTM seeds. **(A)** Characteristic images of rice seed samples at 5 days of germination. Scale bar, 10 mm. **(B)** The time courses showing seeds sample germination rates. **(C)** Characteristic images of seedling emergence at 14 days of germination. Scale bar, 20 mm. **(D)** Seedling emergence rate in **(C)** is presented. **(E)** Characteristic images of rice seedlings. Scale bar, 10 mm. **(F)** Quantification of dry weight of rice seedlings. NT: normal temperature + distilled water treatment; HTM: heat stress + distilled water treatment; HTM + Spd: heat stress + 0.5 mM Spd treatment. Rice plants were treated with Spd solution during 8-12 days after pollination. Heat stress treatment was application at 12-20 days after pollination. Four biological replicates each with 100 seeds for each treatment were set in seed germination and seedling emergence tests. The asterisk (*) or different lowercase(s) above the bars indicate significant differences (p< 0.05, Tukey’s HSD) among treatments.

At 5 and 6 days of germination time, HTM-seeds showed lower germination ability with germination percentage of 31.50% and 55.25% compared with NT. While HTM+Spd seeds germinated much faster than HTM-seeds. Seed germination percentage of HTM+Spd had reached 47.50% and 67.25% at 5 and 6 days of germination, which was 1.51- and 1.23-folds of HTM-seeds, respectively. After 14 days of germination, HTM significantly decreased seedlings establishment rate compared with NT. HTM+Spd treatment showed significant higher seedlings establishment rate compared with HTM (**Figures 2A–D**).

On the other hand, seedling growth was inhibited in HTM seeds, of which seedlings dry weight, seedlings height and total chlorophyll content reached significant levels (**Figures 2E, F**, **Supplementary Figure 2**). On the contrary, the seedlings dry weight, seedlings height, and total chlorophyll content in HTM+Spd were obviously higher than those of HTM. In addition, no significant difference on seed germination rate, seedlings establishment and seedling characteristics were detected between NT and NT+Spd treatments during germination time. After clarifying the role of Spd treatment during seed filling under heat stress in promoting the seed development and subsequent seed germination, only NT, HTM, and HTM+Spd seeds were used in subsequent experiments.

### Spermidine treatment promoted starch synthesis of HTM seeds during rice seed development

**Figure 3:**
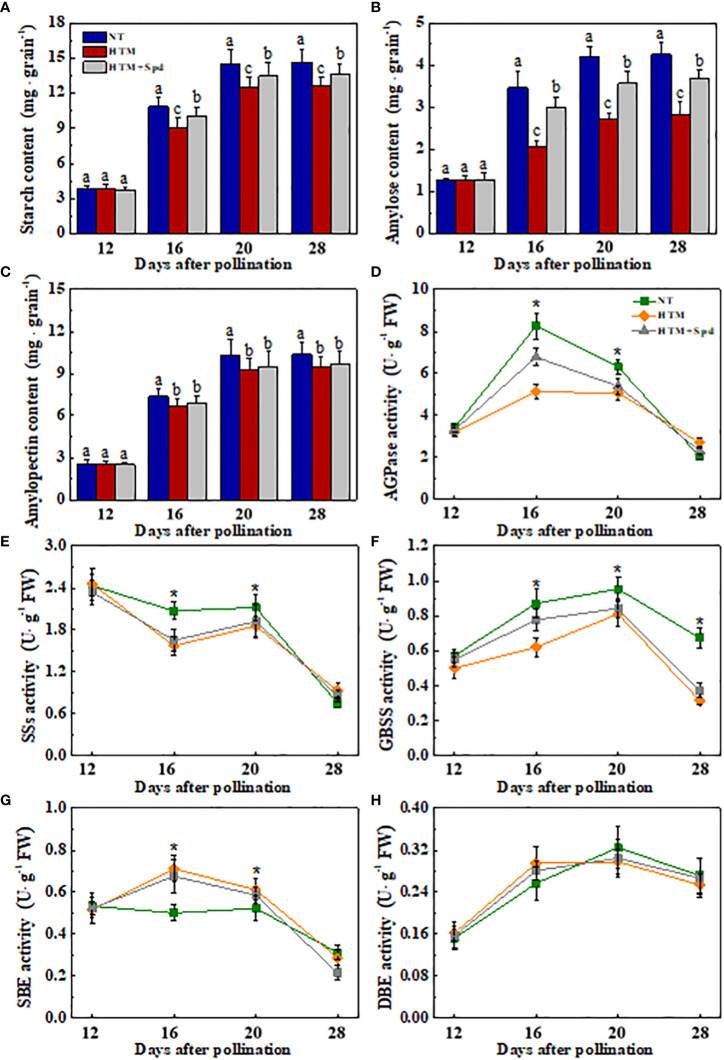
Effect of spermidine treatment on content of starch **(A)**, amylose **(B)**, amylopectin **(C)** and activities of ADPGase **(D)**, SSs **(E)**, GBSS **(F)**, SBE **(G)**, DBE **(H)** in HTM seeds during rice seed development. NT: normal temperature + distilled water treatment; HTM: heat stress + distilled water treatment; HTM + Spd: heat stress + 0.5 mM Spd treatment. AGPase: ADP-glucose pyrophosphorylase; SSs: soluble starch synthases; GBSS: granule-bound starch synthase (GBSS); SBE: starch branching enzyme; DBE: starch debranching enzyme. Rice plants were treated with Spd solution during 8-12 days after pollination. Heat stress treatment was application at 12-20 days after pollination. Results are representative of four independent experiments. The asterisk (*) or different lowercase(s) above the bars indicate significant differences (p< 0.05, Tukey’s HSD) among treatments. .

**Figure 4:**
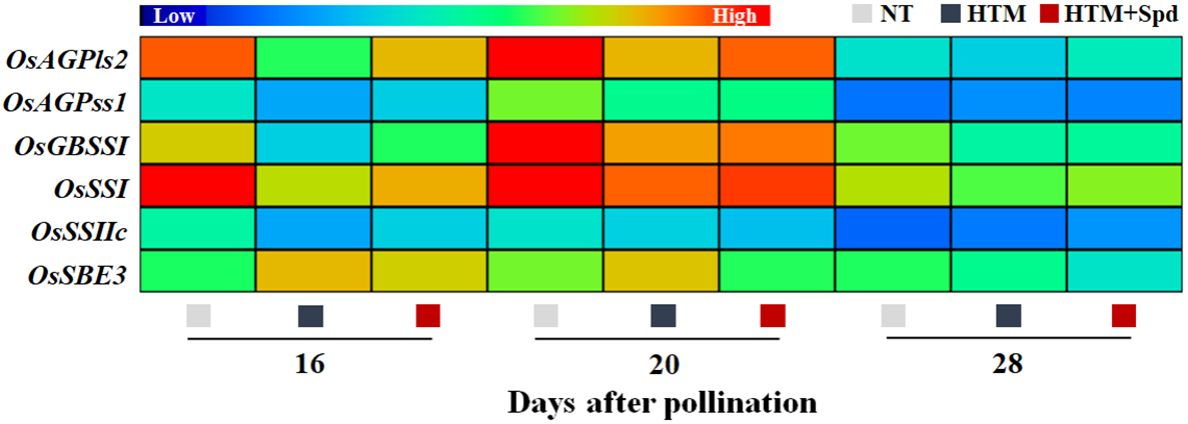
Spd treatment increased the expressions of starch synthesis-related genes in rice seeds during seed development under heat stress. NT, normal temperature + distilled water treatment; HTM, heat stress + distilled water treatment; HTM + Spd, heat stress + 0.5 mM Spd treatment. AGPls, ADP-glucose pyrophosphorylase large subunit; AGPss, ADP-glucose pyrophosphorylase small subunit; GBSS, granule-bound starch synthase; SSs, soluble starch synthases; SBE, starch branching enzyme. RT-qPCR was carried out in three biological replicates, each containing three technical replicates. Heat map was created with Illustrator software. Gene expression from lowest (L) to highest (H) stands for diverse gene levels in the entire database.

It was shown that the contents of total starch, amylase and amylopectin of HTM-seeds were significantly lower than those in NT at 16, 20 and 28 DAP (**Figures 3A–C**). Spd application significantly increased the total starch and amylase contents in HTM seeds at 16, 20 and 28 DAP. While there was no remarkable difference in amylopectin content between HTM and HTM+Spd seeds during seed development. In consistent with the above results, enzymes activity analysis revealed that HTM significantly decreased the activities of AGPase, SSs, and GBSS, and increased SBE activity at 16 and 20 DAP. Besides, HTM+Spd seeds showed significant higher activities of AGPase and GBSS at DAP 16 compared with HTM. The activities of SSs, SBE and DBE were not obviously affected by Spd in HTM seeds (**Figures 3D–H**). The RT-qPCR indicated that the transcripts of starch-synthesis related genes were significantly down-regulated by HTM at 16 and 20 DAP, including *OsAGPls2*, *OsAGPss1*, *OsGBSSI*, *OsSSI*, *OsSSIIc*, and *OsSBE3*. On the contrary, HTM+Spd significantly increased the transcript of *OsAGPls2*, *OsGBSSI*, *OsSSI*, and *OsSBE3* at 16 or 20 DAP compared with HTM (**Figure 4**).

### Spermidine treatment induced antioxidant defense and alleviated excessive ROS during HTM rice seed development

**Figure 5:**
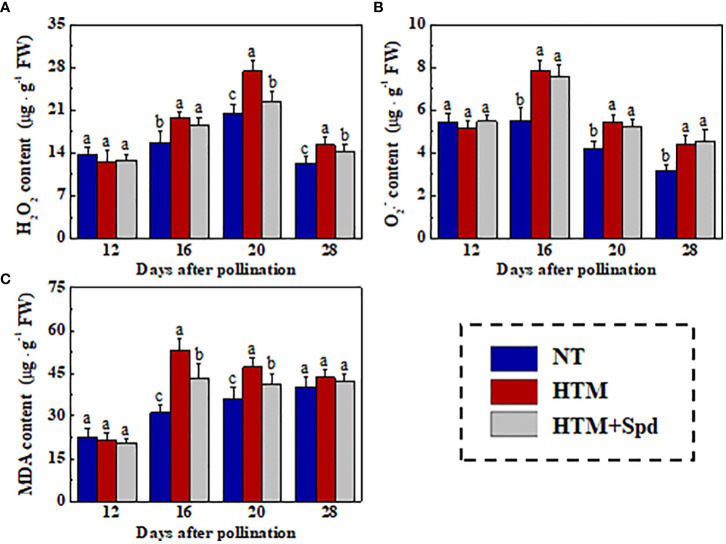
Spd treatment lowered the contents of H_2_O_2_ **(A)**, ${\text{O}}_{2}^{-}$ **(B)**, and MDA **(C)** of HTM rice seeds during seed development. NT, normal temperature + distilled water treatment; HTM, heat stress + distilled water treatment; HTM + Spd, heat stress + 0.5 mM Spd treatment. H_2_O_2_, hydrogen peroxide; ${\text{O}}_{2}^{-}$ , superoxide anion; MDA, malondialdehyde; The determination of H2O2, ${\text{O}}_{2}^{-}$ , and MDA were performed with four biological replicates. Diverse lowercase letters stand for significant differences across treatments (p< 0.05, Tukey’s HSD).

The excessive MDA and ROS contents in rice seeds induced by heat stress were the critical causes of the decreased seed vigor. For further investigated the mechanism of Spd in improving germination and seedling establishment of HTM rice seeds, we determined the H_2_O_2_, ${\text{O}}_{2}^{-}$ , and MDA contents in rice seeds during seed development (**Figure 5**). HTM significantly increased the H_2_O_2_, ${\text{O}}_{2}^{-}$ , and MDA contents at 16 and 20 DAP. By contrast, the contents of H_2_O_2_ (20 and 28 DAP) and MDA (16 and 20 DAP) in HTM+Spd seeds were apparently lower than those in HTM seeds. However, exogenous Spd did not significantly affect ${\text{O}}_{2}^{-}$ level in HTM seeds at 16, 20, 28 DAP.

**Figure 6:**
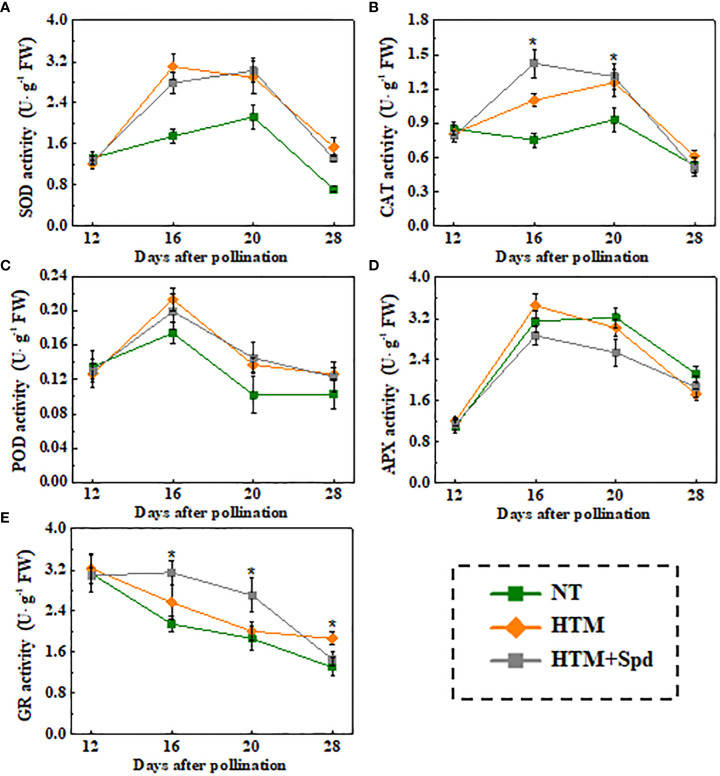
Spd treatment enhanced SOD **(A)**, CAT **(B)**, POD **(C)**, APX **(D)**, and GR **(E)** activities of HTM rice seeds during seed development. NT, normal temperature + distilled water treatment; HTM, heat stress + distilled water treatment; HTM + Spd, heat stress + 0.5 mM Spd treatment. SOD, superoxide dismutase; CAT, catalase; POD, peroxidase; APX, ascorbate peroxidase; GR, glutathione reductase. The enzymes activity analysis was performed with four biological replicates. The asterisk (*) indicates significant differences (p< 0.05, Tukey’s HSD) across treatments.

**Figure 7:**
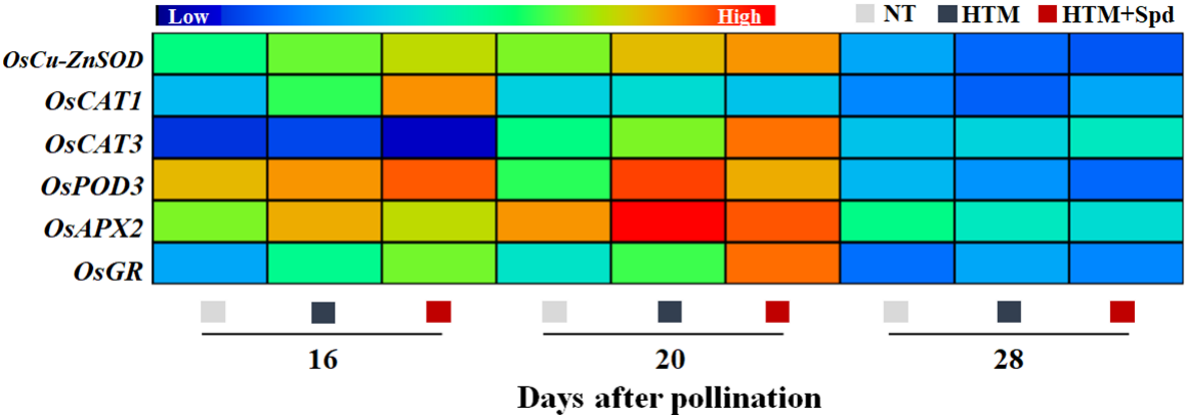
Spd treatment up-regulated the expressions of antioxidant enzyme genes in HTM seeds during seed development. NT, normal temperature + distilled water treatment; HTM, heat stress + distilled water treatment; HTM + Spd, heat stress + 0.5 mM Spd treatment. SOD, superoxide dismutase; CAT, catalase; POD, peroxidase; APX, ascorbate peroxidase; GR, glutathione reductase. RT-qPCR was carried out in three biological replicates, each containing three technical replicates. Heat map was made with Illustrator software. Gene expression from lowest (L) to highest (H) stands for diverse gene levels in the entire database.

Given that Spd treatment decreased contents of H_2_O_2_ and MDA in HTM seeds during seed development, the Spd role in antioxidant enzyme activities, including SOD, CAT, OPD, APX, and GR, was examined (**Figure 6**). As a result, HTM apparently enhanced SOD, CAT, and POD activities at 16 and 20 DAP compared with NT seeds. Besides, Spd remarkably increased the CAT (16 DAP) and GR (16 and 20 DAP) activities in HTM seeds. While no significant effect of Spd on SOD, POD activities was observed in HTM seeds during seed development. Consistent with the above results, RT-qPCR showed that the transcriptional levels of antioxidant enzyme related-genes were markedly up-regulated by HTM, such as *OsCu-ZnSOD*, *OsCAT1*, *OsCAT3*, *OsPOD3*, *OsAPX2*, and *OsGR* (**Figure 7**). Besides, significantly higher transcriptional levels of *OsCu-ZnSOD, OsCAT1*, *OsCAT3*, *OsGR* were observed in HTM+Spd seeds compared with HTM seeds. However, it was shown that Spd decreased the transcriptional levels of *OsPOD3* and *OsAPX2* at 16 and 20 DAP.

### Spd treatment improved starch hydrolysis in HTM rice seeds during early germination time

**Figure 8:**
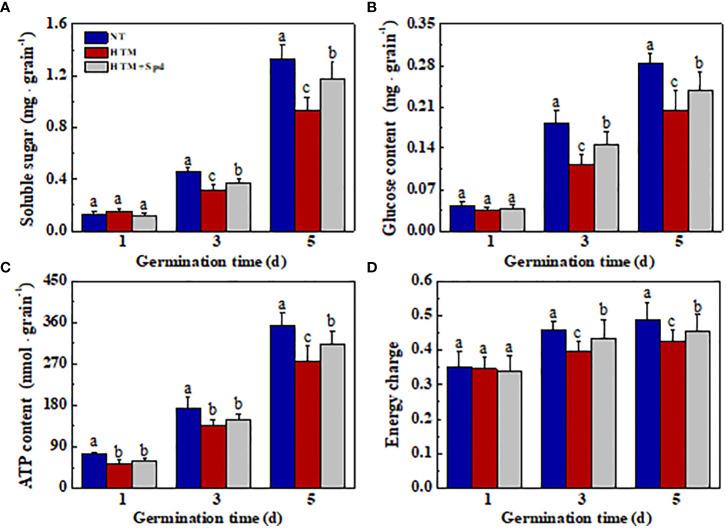
Spermidine treatment during rice seed development under heat stress increased contents of soluble sugar **(A)**, glucose **(B)**, ATP **(C)**, and energy charge **(D)** in rice seeds during germination time. NT, normal temperature + distilled water treatment; HTM, heat stress + distilled water treatment; HTM + Spd, heat stress + 0.5 mM Spd treatment. Percentages stand for averages of four tests ± SE. Different lowercase(s) above the bars stand for statistical significance (p<0.05, Tukey’s HSD) among treatments.

Seed storage substances are the main energy sources in early seed germination and seedling emergence, while soluble sugar is the major nutrient generated via storage substance decomposition. For better analyzing the mechanism by which Spd promoted germination and seedling emergence from HTM seeds, the levels of soluble sugar, glucose, ATP, and energy charge were determined during early germination (**Figure 8**). Compared with NT, HTM remarkably lowed the levels of soluble sugar, glucose, ATP and energy charge at 3 and 5 days of germination. In contrast, Spd treatment remarkably increased the contents of soluble sugar and glucose in HTM seeds at 3 and 5 days of germination. Besides, ATP and energy charge levels apparently increased in HTM+Spd seeds at 3 and 5 days of germination relative to HTM seeds. The above results suggested that Spd treatment promoted germination and seedling emergence of HTM seeds by increasing ATP and soluble sugar contents.

**Figure 9:**
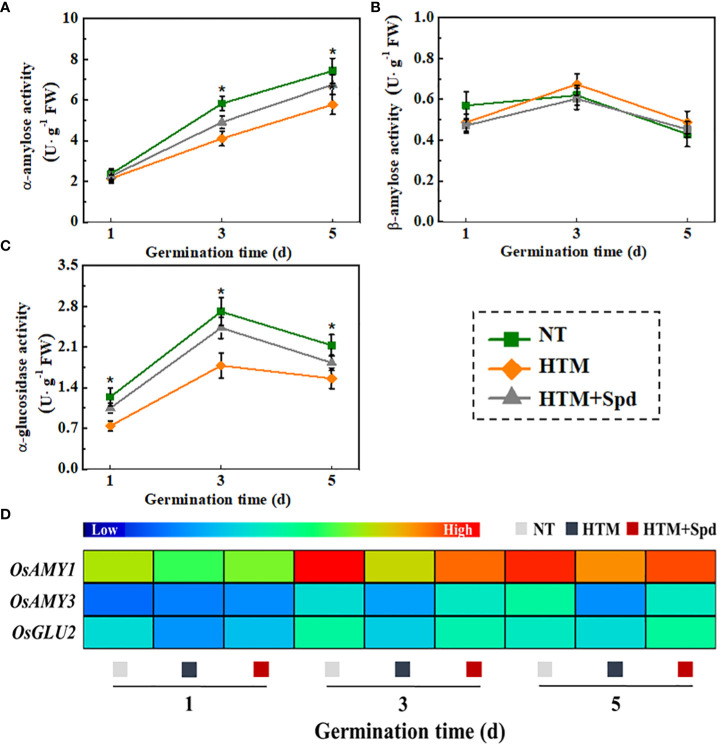
Spermidine treatment increased the activities of α-amylose **(A)**, β-amylose **(B)**, α-glucosidase **(C)** and expressions of starch metabolism-related genes **(D)**. NT, normal temperature + distilled water treatment; HTM, heat stress + distilled water treatment; HTM + Spd, heat stress + 0.5 mM Spd treatment. Four biological replicates for each treatment were set in enzymes activity assay. Realtime quantitative PCR was performed with three biological replications, and each was made in three technical replicates. The asterisk (*) was indicative of significant differences (p<0.05, Tukey’s HSD) across treatments. The Illustrator software was used for creating the heat map. The gene levels from low (L) to high (H) indicated the lowest and highest levels in the whole database.

During seed germination and early seedling emergence, starch is hydrolyzed by α-amylose and β-amylose to produce maltose, which then converted into glucose through α-glucosidase. In present study, soluble sugar content elevated in HTM+Spd seeds, and its underlying mechanism was explored by measuring the activities of α-amylose, β-amylose, and α-glucosidase (**Figures 9A–C**). It was shown that HTM significantly lowered the activities of α-amylose and α-glucosidase during rice seeds germination. By contrast, remarkably up-regulated activities of α-amylose and α-glucosidase were observed in HTM+Spd seeds compared with HTM seeds. However, no significant difference of β-amylose activity was detected between three treatments. These observations conformed to positive role of Spd in soluble sugar and glucose contents during early seed germination.

Additionally, significant lower transcriptional levels of *OsAmy1, OsAmy3, OsGlu2* were detected in HTM seeds at 1, 3, 5 DAP compared with NT seeds (**Figure 9D**). It was worth noting that Spd application up-regulated the transcriptional levels of *OsAmy1*, *OsAmy3* in HTM seeds at 3 and 5 days of germination. In addition, significant higher transcriptional level of *OsGlu2* was detected in HTM+Spd seeds at 1 and 3 days of germination. Such results consistent with the role of Spd in α-amylose and α-glucosidase activities of HTM+Spd rice seeds during germination.

### Role of Spd in GA and ABA levels in HTM rice seeds in early germination

**Figure 10:**
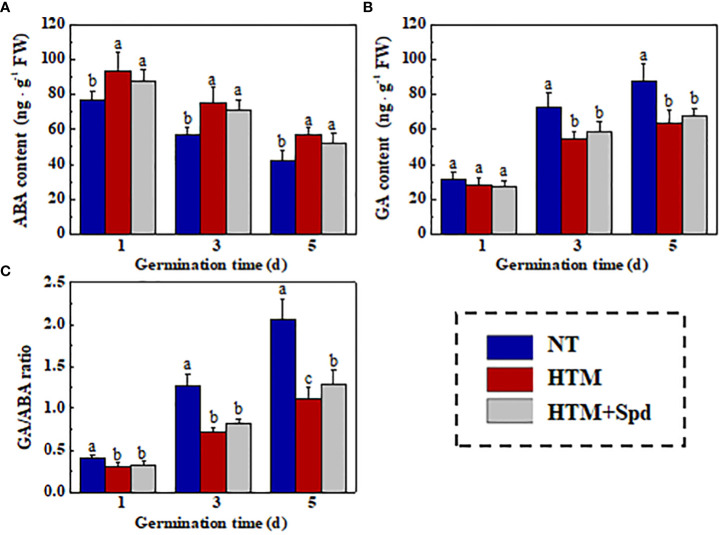
The effect of spermidine treatment on contents of ABA **(A)**, GA **(B)**, and GA/ABA ratio **(C)** in HTM seed during early germination time. NT, normal temperature + distilled water treatment; HTM, heat stress + distilled water treatment; HTM + Spd, heat stress + 0.5 mM Spd treatment. Percentages indicate the averages of four tests ± SE. Different lowercase(s) above the bars stand for statistical significance (p<0.05, Tukey’s HSD) among treatments.

As gibberellin (GA) and abscisic acid (ABA) are important for the seed germination (Shu et al., 2016; Shu et al., 2018), we further analyzed the relation of Spd role in rice seed heat tolerance with GA/ABA pathways in rice seed germination (**Figure 10**). It was shown that ABA content slightly declined, whereas GA content evidently elevated in NT seeds during early germination. HTM significantly enhanced the ABA level and lowered the GA level at 3 and 5 days of germination, resulting in significant lower GA/ABA ratio at 1, 3, 5 DAP. However, the ABA, GA contents and GA/ABA ratio in HTM+Spd seeds showed no significant difference with those in HTM seeds during seed germination.

## Discussion

Rice plants are vulnerable to heat stress during grain filling process, which seriously affect the seed yield and quality (Peng et al., 2004; Zhao et al., 2017; Yang et al., 2020). However, most studies on the rice heat tolerance focused on the roles of endogenous or environmental cues specifically during flowering, pollination and early grain filling stages (Madan et al., 2012; Fu et al., 2019; Chen et al., 2021). Although some reports had been published on the effects of heat stress during mid or late seed filling stages on the seed development (Morita et al., 2005; Beáta et al., 2008; Lin et al., 2010), the detailed regulatory mechanism remains largely unknown, especially the role of exogenous substances to rice heat tolerance during seed mid-filling stage on subsequent seed germination. According to our results, HTM remarkably suppressed the seed development, germination and seedling establishment. Spd treatment markedly mitigated heat injury, improved HTM rice seed germination and seedling establishment.

### Spd alleviated the heat damage of HTM on rice seed development

Several reports found that heat stress during rice pollination and early grain filling stage remarkably decreased the seed maturing rate, seed dry weight, and seed size (Tang et al., 2017; Zhang, 2022). Seed size and shape are determined by endosperm cell number and size. It was reported that heat stress (34°C) decreased cell size midway between endosperm surface and central point, resulting in the reduce of rice seed filling rate and seed thickness (Morita et al., 2005). Similarly, the present study found that HTM significantly suppressed rice seed development, which manifested as the lower seed width, seed length, seed volume and dry weight. However, HTM did not dramatically influence the seed thickness. Fu et al. (2019) found that high temperature treatment in the early grain filling stage (7-11 DAP) evidently decreased the rice seed thickness, but not significantly affected seed length or width. It was proposed that the impact of high temperature on rice seed size varied depending on the treatment period and rice variety. It was noting that Spd application notably improved the length, width and dry weight of HTM seeds. Consistently, several studies revealed a similar effect of Spd application on seed heat tolerance during blooming or early filling stage (Fu et al., 2019; Chen et al., 2021).

### Spd effectively promoted the germination and seedling establishment of HTM rice seeds

Certain reports investigated the effect of parental environmental factors in subsequent seed germination (Nonogaki et al., 2014; Postma and Agren, 2015; Awan et al., 2018), while the detailed regulatory mechanism is still unknown. Brunel-Muguet et al. (2016) reported that oilseed rape seeds development under heat stress (33°C/day, 19°C/night) during seed filling are associated with high sprouting rate after harvesting, reduced ABA level and high aberrant seedling rate. In present study, HTM remarkably lowered rice seed germination speed and decreased the seedlings dry weight, seedlings height and total chlorophyll content. By contrast, Spd application effectively promoted seed germination and seedling growth in HTM seeds. HTM+Spd treatment showed significant higher seed germination rate, seedlings establishment rate, and seedlings characteristics compared with HTM. Chen et al. (2021) found that 1.5 mM Spd treatment remarkably enhanced rice seed germination index in the early filling stage upon heat stress condition, while *OsSAP5* was the potentially important gene related to heart resistance of rice treated by Spd. Therefore, Spd possibly has a critical effect on the evolution of rice seed vigor upon heat stress conditions in diverse seed filling stages.

### Spd promoted the starch synthesis of HTM rice seeds during seed development

Several studies explored the adverse impacts of heat stress on starch synthesis during crops seed development (Su et al., 2009; Lin et al., 2010; Yamakawa and Hakata, 2010; Sreenivasulu et al., 2015). Consistently, our results revealed that HTM significantly decreased contents of amylose, amylopectin, and total starch through down-regulating the activities of several key starch biosynthesis enzymes and corresponding-genes expressions. Spd was proved to be involved in starch metabolism. It was found that Spd treatment remarkably enhanced starch content in wheat grains during post-anthesis process under drought stress (Yang et al., 2014). Wang et al. (2012) found that starch accumulation rate was positively correlated with Spd content in superior and inferior spikelets of rice. Herein, Spd application efficiently increased AGPase and GBSS activities and transcripts of corresponding-genes expressions (OsAGPls2, OsAGPss1, and OsGBSSI), resulting in significant higher contents of starch and amylose in HTM seeds. While the amylopectin content was not significantly affected by Spd in rice seeds under heat stress. Consistent with the above results, Fu et al. (2019) found that the Spd-treated rice seeds showed improved seed development with higher amylose level under heat stress during early seed development. It was suggested that the effect of Spd on starch accumulation response to heat stress at different stages of rice seed development was consistent. Moreover, it was proved that amylose level made greater effects on seed germination compared with total starch and amylopectin, and high vigor hybrid rice seeds always showed higher amylose content and lower amylopectin content (Zhang, 2014). It was speculated that amylose plays a critical role in the role of Spd in improving heat tolerance of rice seeds during filling stage.

### Spd induced the antioxidant defense and alleviated the over-accumulation of H_2_O_2_ and MAD in HTM rice seeds during seed development

Reactive oxygen species (ROS) mainly including hydrogen peroxide (H_2_O_2_), superoxide anion ( ${\text{O}}_{2}^{-}$ ) and hydroxyl radical (OH)(Sachdev et al., 2021). ROS exert dual functions, to be specific, ROS at the suitable levels can positively affect seed development, germination, and environmental stress resistance, while excessive ROS contents generated toxicity to plant cells and tissues (Barba-Espin et al., 2011; Oracz and Karpinski, 2016; Sachdev et al., 2021). Consistently, we found HTM induced overaccumulation of H_2_O_2_, ${\text{O}}_{2}^{-}$ , and MDA in rice seed during mid-filling stage (**Figure 5**). HTM+Spd markedly mitigated the excessive MAD and H_2_O_2_ contents at 16 and 20 DAP. It was suggested that maintaining ROS homeostasis in seed development process is of great significance for guaranteeing the evolution of seed vigor (Rajjou et al., 2012; Rashid et al., 2020). Our results revealed that Spd application could enable better ROS scavenging ability in rice seeds during mid-filling stage under heat stress. Such results were consistent with prior findings that maintenance of ROS homeostasis accounts for a mechanism related to Spd in improving seed vigor during seed filling or early germination upon several abiotic stresses (Pan et al., 2014; Yang et al., 2014; Sheteiwy et al., 2017; Tao et al., 2018).

Antioxidases, mainly including SOD, POD, CAT, APX, and GR, are crucial to the ROS scavenging system (Sachdev et al., 2021). POD activity showed positive relation to seed vigor index, whereas MDA level displayed negative relation (Cui et al., 2014). Exogenous Spd could eliminates ROS through enhancing CAT, SOD and POD activities in tomato cells under upon stress conditions (Diao et al., 2015). During rice flowering period, Spd-treated leaves exhibited reduced MDA level and enhanced SOD and POD activities (Tang et al., 2018). Likewise, this study suggested that activities of SOD, CAT, POD, GR and related genes expressions were enhanced by HTM. In addition, HTM+Spd seeds displayed remarkably enhanced SOD, CAT, and GR activities and corresponding-genes (including OsCu-ZnSOD1, OsCAT1, OsCAT3, and OsGR) expressions. CAT functions in the decompose of H_2_O_2_ to water and oxygen, while SOD could disproportion superoxide anion free radicals for producing H_2_O_2_ and oxygen (Sachdev et al., 2021). In addition, GR is responsible for the reduction of oxidaized glutathione disulfide to reduced glutathione, which provides reducing power for ROS scavenging (Smiri et al., 2010). Our results conformed to prior research suggesting that Spd protected macromolecules and biofilms and maintained organelle integrity upon stresses (Diao et al., 2015). The positive role of Spd on activating of antioxidant defense was also proved by several previous studies (Liu et al., 2014; Li et al., 2016; Zhang YP et al., 2017; Sang et al., 2017).

### Spd promoted the starch degradation during early germination and improved the energy supply of HTM rice seeds

Starch is the main storage polysaccharide in rice seeds (Gorim and Asch, 2014; Kaneko et al., 2004). Starch level is tightly linked to rice seed vigor and germination (Kim et al., 2006). The present results showed that HTM resulted in significant lower starch content and poor seed germination; while Spd application improved the seed germination of HTM seeds. However, whether starch degradation was crucial to the improvement of Spd on seed germination was unclear. Rather than β-amylose, α-amylase and α-glucosidase were proved to be closely related with seed germination in rice and maize (Zhao and Wang, 2001). α-amylase and α-glucosidase are mostly detected in cells of aleurone layer and scutellar epithelium in germinated cereal seeds (Gorim and Asch, 2014; Kaneko et al., 2004). α-amylase is secreted via aleurone layer and released in endosperm for catalyzing stored starch hydrolysis into maltose and maltotriose, which were then converted to glucose by α-glucosidase (Catusse et al., 2011). It is well known that the ATP supplied by glycolytic process is crucial to support seed germination and seedling establishment (Rajjou et al., 2012). Our results revealed that HTM resulted in inhibitory effects on the induction of α-amylase and α-glucosidase activities, soluble sugar, glucose and ATP content, consequently, the germination of rice seeds at early imbibition time. While Spd application during seed development could alleviated the inhibitory effects of HTM on starch degradation and subsequent seed germination.

GA and ABA represent the important phytohormones related to seed germination and seedling establishment (An and Lin, 2011; Boccaccini et al., 2016; Wang et al., 2021). To be specific, GA contributes to breaking seed dormancy and inducing seed germination, while the high ABA content caused seed dormancy and suppressing seed germination (Jia et al., 2012; Arc et al., 2013). However, weather the GA and ABA pathways were related to the regulation of Spd on starch hydrolase and germination in HTM seeds remain poorly understood. In this study, HTM was found to decrease GA level and increased ABA level in rice seeds during early germination. However, it was unexpected that Spd made no significant effect on GA and ABA level in germinated HTM rice seeds. Typically, GA and ABA metabolism is possibly modulated via signals other than Spd in rice seed resistance to heat stress. GA facilitated seed dormancy breaking and seed germination induction by activating the α-amylose and α-glucosidase activities from aleurone layer, thereby promoting the decomposition of stored starch (Zhang et al., 2011; Liu et al., 2016). It was proposed that the GA metabolism might not be involved in the improvement of Spd on starch hydrolysis during HTM seed germination.

## Conclusion

**Figure 11:**
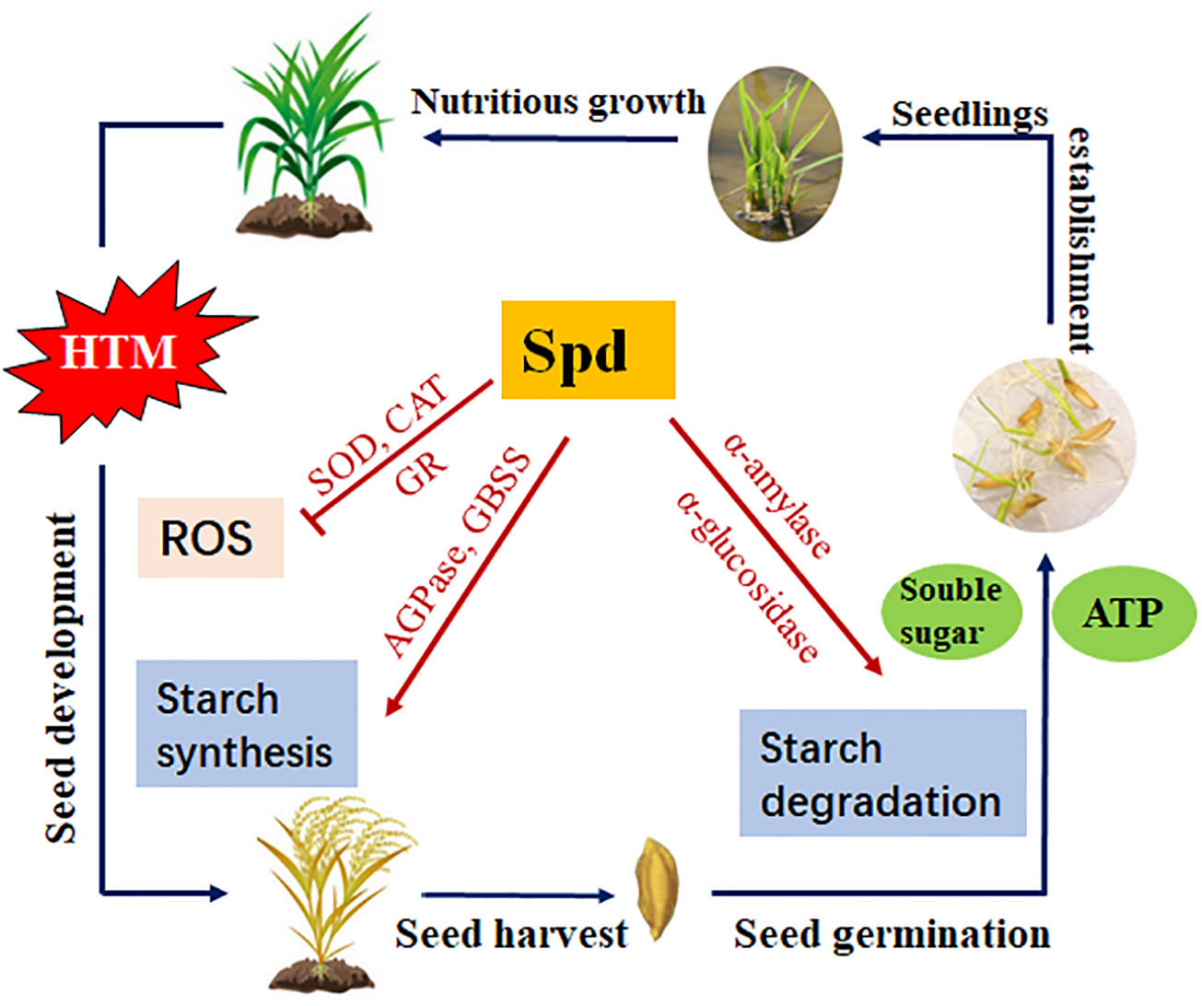
Proposed scheme for the role of Spd in heat tolerance of rice seeds during mid-filling stage and subsequent seed germination. HTM, heat stress treatment during mid-filling stage (12-20 days after pollination); Spd, spermidine; ROS, reactive oxygen species; SOD, superoxide dismutase; CAT, catalase; GR, glutathione reductase; AGPase, ADP-glucose pyrophosphorylase; GBSS, granule-bound starch synthase.

In summary, the present study revealed that the excess ROS levels, block of starch biosynthesis during seed filling, as well as the inhibited amylohydrolysis pathway and metabolic imbalance of GA/ABA, might be the potential causes of deterioration vigor of HTM rice seeds. Spd treatment markedly mitigated heat injury, improved HTM rice seed germination and seedling establishment, and this was possibly closely related to antioxidant defense and starch metabolism (**Figure 11**). This work sheds more lights on the theoretical and practical foundation for the application of Spd in enhancing the production of crop seeds.

## Data availability statement

The original contributions presented in the study are included in the article/**Supplementary Material**. Further inquiries can be directed to the corresponding author.

## Author contributions

Conceptualization, YH and YQ; Investigation, YH and GM; resources, YH and YQ; writing-original draft preparation, YH and LY; writing-review and editing, YQ; supervision, DC, LY, and XR; All authors contributed to the article and approved the submitted version.
