# Supplementary material for: Spermidine enhances heat tolerance of rice seeds during mid-filling stage and promote subsequent seed germination
Source: Front Plant Sci. 2023 Sep 18;14:1230331. doi: 10.3389/fpls.2023.1230331 (PMC10543890; doi:10.3389/fpls.2023.1230331)
Supplement: Supplementary file 1 [file DataSheet_1.docx]

**Spermidine enhances heat tolerance of rice seeds during mid-filling stage and promote subsequent seed germination**

**Table S1.** Primers used in qRT-PCR analysis of genes expression

| **Gene name** | **Primer sequence** | |
| --- | --- | --- |
| *18s rRNA* | F: 5´- CTACGTCCCTGCCCTTTGTACA -3´ | R: 5´- ACACTTCACCGGACCATTCAA -3´ |
| *OsAGPls2* | F: 5´- TTCCTCTCACAAGCACAAGG -3´ | R: 5´- AAACAGTTGCTCATGGGGATA -3´ |
| *OsAGPss1* | F: 5´- CGAGGGCCTATGGAAACAA -3´ | R: 5´- CAGCATCTGCAGTACCCTGA -3´ |
| *OsGBSSI* | F: 5´- GATTTCATCGACGGGTATGAG -3´ | R: 5´- ATTCCGGCCTTCATCCAG -3´ |
| *OsSSI* | F: 5´- TGCTCCTTGGTCCAAAACA -3´ | R: 5´- TGACCTCTCCTAGCCAAAGC -3´ |
| *OsSSIIc* | F: 5´- TGCAGGTGCTTTACCCAAA -3´ | F: 5´- GCGTAATCACCGTACTTGG -3´ |
| *OsSBE3* | F: 5´- TGGTTTAGTTGTGCTCATGGAT -3´ | R: 5´- CAAAACCGTTCAACCCATCT -3´ |
| *OsCu-ZnSOD* | F: 5´- ATCCACTCCTTTGGCGACAC -3´ | R: 5´- AATGAGGCCCGCTTAGTGAA-3´ |
| *OsCAT1* | F: 5´- ACCAAACTACCTGCTGCTCC -3´ | R: 5´- GATCGTAGCGGGATGGGAAG-3´ |
| *OsCAT3* | F: 5´- GGACGAGGAGGTGGACTACT-3´ | R: 5´- TGCTTGTGTATCGTCGCCTT-3´ |
| *OsPOD3* | F: 5´- CGTCCATCCTCCTCAACTCG -3´ | R: 5´- GGTCGATGAGGTCGAACCCT -3´ |
| *OsAPX2* | F: 5´- CTTGTGAGTGGCGAGAAGGA -3´ | R: 5´- AAAGAAGGCGTCCTCATCCG-3´ |
| *OsGR* | F: 5´- GGACGTGTGTGATACGTGGT -3´ | R: 5´- CCCCATTGATTTCCCACCCA -3´ |
| *OsAMY1* | F: 5´- CCATGCATCTTCTACGACCA -3´ | R: 5´- TCCCGTTCTGGTCTCTGATT-3´ |
| *OsAMY3* | F: 5´- AGAGGTCAACGAGCGAAGAA -3´ | R: 5´- TGCCGCTAACCTCTCAATTT -3´ |
| *OsGLU2* | F: 5´-ATGTTTGGTGCACTGGGGAA -3´ | R: 5´-CACCTCGTACTGGTGGCATA -3´ |

**Fig.S1.** **Seed weight of rice during seed development process**


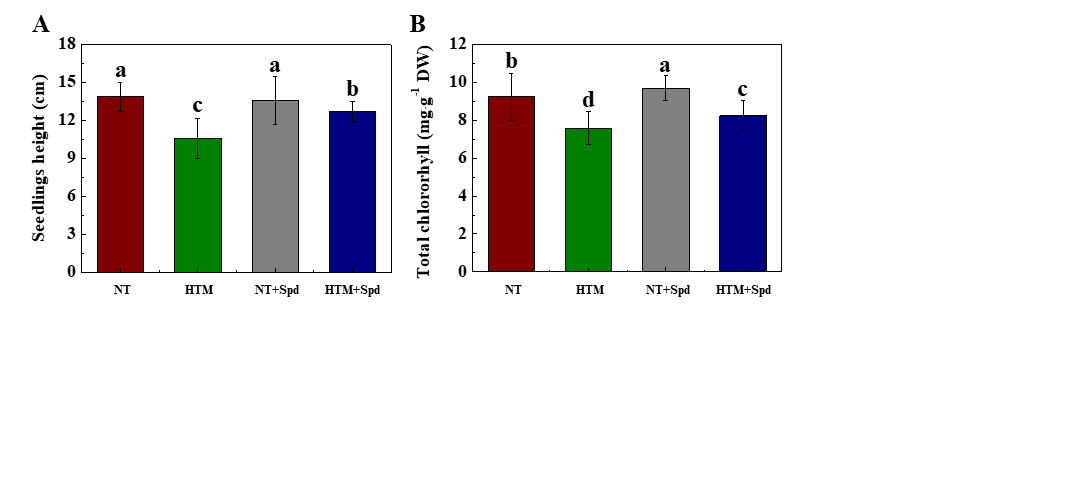


**Fig.S2.** Spermidine treatment increased seedling height (A) and seedlings total chlorophyll content (B) from HTM rice seeds.

NT: normal temperature + distilled water treatment; HTM: heat stress + distilled water treatment; HTM+Spd: heat stress +1.5 mM Spd treatment. Rice plants were treated with Spd solution during 8-12 days after pollination. Heat stress treatment was application at 12-20 days after pollination. Different lowercase(s) above the bars indicate significant differences (p < 0.05, Tukey’s HSD) among treatments.
